# Association between ZJU index and glycemic outcomes in individuals with impaired fasting glucose: a retrospective multicenter Chinese cohort study

**DOI:** 10.3389/fendo.2026.1811341

**Published:** 2026-04-23

**Authors:** Wanlan Huang, Duo Yang, Renzhe Lin, Sen Li, Shujun Ye, Zitian Luo, Huankai Zhang, Si Wu, Longsheng Zhang

**Affiliations:** 1 Department of Oncology, Jieyang People’s Hospital, Jieyang, Guangdong, China; 2 Department of Anesthesiology, Jieyang People’s Hospital, Jieyang, Guangdong, China; 3 First Clinical Medical College, Guangdong Medical University, Zhanjiang, Guangdong, China

**Keywords:** diabetes, endocrinology, glycemic reversion, impaired fasting glucose, insulin resistance, prediabetes, risk stratification, ZJU index

## Abstract

**Background:**

DM is a chronic metabolic disorder with a globally increasing prevalence. IFG, a major subtype of prediabetes, is highly prevalent amongthe Chinese adult population and represents a critical window for therapeutic intervention. The ZJU index is a comprehensive metabolic indicator that reflects systemic insulin resistance and metabolic burden status. However, its association with bidirectional glycemic outcomes specifically in individuals with IFG remains unclear. This study aimed to investigate the impact of the baseline ZJU index on the progression to diabetes, as well as on the reversion to normoglycemia.

**Methods:**

This multicenter retrospective cohort study analyzed data from 11,243 individuals with IFG enrolled in a health screening program between 2010 and 2016. The ZJU index, calculated using a standardized formula, served as the primary exposure variable. We employed Cox proportional hazards regression and logistic regression models to examine the association between the ZJU index and glycemic status transition. RCS was used to explore nonlinear relationships, and a threshold effect analysis was conducted to identify potential inflection points. Subgroup and sensitivity analyses were performed to assess the robustness of the associations.

**Results:**

Higher baseline ZJU index quartiles were significantly associated with a decreased likelihood of reversion to normoglycemia and an increased risk of diabetes progression. In the fully adjusted Cox model, each one-unit increase in the ZJU index was associated with a 3% lower hazard of reversion to normoglycemia and a 12% higher hazard of progression to DM. RCS analysis revealed a linear inverse association with reversion and a nonlinear positive association with progression, with an inflection point at a ZJU index of 38.499. Subgroup and sensitivity analyses confirmed the robustness and consistency of these associations across diverse population strata and data handling methods.

**Conclusions:**

In individuals with IFG, a higher ZJU index demonstrates​ a linear inverse association with reversion to normoglycemia and a non-linear positive association with progression to incident DM. Accordingly, the ZJU index can serve as​ a practical tool for risk stratification and precision management, guiding preventive strategies for those at high risk of DM.

## Background

Diabetes mellitus (DM) and its complications have emerged as a significant global public health challenge. According to recent estimates, approximately 589 million adults worldwide were living with DM in 2024, corresponding to a global prevalence of 11.11%. The prevalence of diabetes is projected to rise to 12.96%, affecting 853 million adults by 2050. The disease burden is unevenly distributed, with prevalence peaking in adults aged 75–79 years and being generally higher in males and in urban populations (1, 2). China has the world’s largest population of individuals with DM, exceeding 118 million, which accounts for about 22% of the global total (3, 4). The country ranks first in the world in terms of the number of DM cases, and there exists a substantial number of undiagnosed individuals, making the prevention and control situation extremely severe. Impaired fasting glucose (IFG) is a key state of prediabetes and is considered an inevitable transitional stage from normal glucose homeostasis to DM. The latest IDF data indicates that approximately 487.7 million adults globally have IFG, and this number is projected to rise to 647.5 million by 2050 (5). As a country with a high burden of DM, China faces a particularly heavy disease burden from prediabetes. Recent epidemiological surveys show that the prevalence of prediabetes among Chinese adults is as high as 38.1%, with IFG being one of the main subtypes (6). As a “prelude” and critical window period for the development of DM, the IFG stage is not only closely associated with an increased risk of cardiovascular disease but, more importantly, exhibits significant heterogeneity in its glycemic outcomes (7). Research indicates that IFG does not inevitably progress to DM. In the natural course, although about 5%-10% of individuals with IFG progress to DM annually, a considerable proportion (20%-50%) can successfully revert to normoglycemia (7, 8), depending on various prognostic factors. This “reversion” phenomenon holds high clinical value because, once normoglycemia is restored, the patient’s risk of cardiovascular events and all-cause mortality significantly decreases (8). However, there is currently a lack of simple, easy-to-use, and efficient risk stratification tools in clinical practice to early identify this “reversible” advantageous population or to identify high-risk individuals prone to progression (6, 9). Existing assessment methods mostly rely on single indicators such as blood glucose or glycated hemoglobin A1c (HbA1c), which often fail to capture the complex metabolic heterogeneity underlying the IFG population, leading to an inability to achieve precise risk-stratified management (10). Therefore, exploring novel biomarkers capable of integrating multi-dimensional metabolic information is of urgent clinical significance for guiding early intervention and risk assessment in the IFG population (11).

The development of IFG is closely associated with insulin resistance (IR) (12). In conditions of visceral obesity and dyslipidemia, excess free fatty acids flow to the liver, leading to intrahepatic ectopic fat deposition, which subsequently impairs the ability of insulin to suppress hepatic glucose production, ultimately resulting in elevated fasting glucose (12, 13). This process indicates that IFG is not merely a singular glycemic abnormality but rather a complex interactive network involving “obesity-adipose tissue dysfunction-hepatic metabolic disturbance”. However, currently used single clinical indicators have obvious limitations in evaluating this complex “obesity-adipose tissue dysfunction-hepatic metabolic disturbance” network. For instance, although fasting plasma glucose (FPG) is the diagnostic criterion for IFG, it primarily reflects an instantaneous glycemic state and fails to capture underlying lipotoxicity or hepatic metabolic burden (14). Similarly, body mass index (BMI), while reflecting overall obesity, cannot distinguish visceral fat from subcutaneous fat (15). Although the hyperinsulinemic-euglycemic clamp technique is the gold standard for assessing IR, it is cumbersome, expensive, and invasive, making it unsuitable for large-scale population screening or routine clinical monitoring (16). Therefore, developing non-invasive composite surrogate indices that can integrate multidimensional information encompassing obesity, glucose/lipid metabolism, and hepatic function has become a current research focus in metabolic diseases, holding significant translational medical value for the early identification of high-risk IFG individuals (16, 17).

Chinese researchers led by Wang et al. developed a novel comprehensive metabolic index named the Zhejiang University Index (ZJU index) (18). The construction of the ZJU index was based on a large-scale population cohort study. Its calculation formula integrates BMI, FPG, triglycerides (TG), and the alanine aminotransferase to aspartate aminotransferase ratio (ALT/AST ratio) (18, 19). This multi-dimensional design makes it more than just an obesity indicator; it is a composite scoring system capable of simultaneously reflecting systemic obesity burden, glucose/lipid metabolism dysregulation, and the degree of hepatocellular injury. Existing research has sufficiently demonstrated the value of the ZJU index in metabolic disease management. In a 2025 editorial, Gouda et al. pointed out that the ZJU index was significantly superior to traditional models such as the fatty liver index (FLI) and hepatic steatosis index (HSI) in predicting the risk and severity of metabolic dysfunction-associated steatotic liver disease (MASLD), particularly excelling in identifying Asian individuals with metabolic abnormalities (20). Multiple studies have shown that the ZJU index is highly positively correlated with the level of IR and can serve as an effective surrogate biomarker for assessing the severity of IR (21, 22). A longitudinal cohort study published by Wu et al. in 2024, involving over 15000 participants, was the first to confirm that an elevated baseline ZJU index was independently associated with an increased risk of incident DM in the general population, demonstrating a significant dose-response relationship (22). Compared to other surrogate indices such as the TyG index or visceral adiposity index (VAI), the unique advantage of the ZJU index lies in its inclusion of the ALT/AST ratio (19, 22). Alterations in liver enzyme ratios typically precede overt structural liver pathology and can sensitively reflect impaired hepatic insulin sensitivity and early metabolic stress. Given that the core pathophysiological feature of the IFG population is precisely hepatic IR, the design of the ZJU index—which highlights the “central role of the liver in metabolic regulation”—may endow it with unique pathophysiological advantages and clinical potential for stratifying the risk of heterogeneous outcomes in the IFG population.

Although the ZJU index has demonstrated promising potential in metabolic disease risk assessment, its current evidence base remains limited and requires further investigation. A review of the existing literature reveals that the vast majority of studies have focused on two population types: first, assessing the association between the ZJU index and incident metabolic diseases (such as DM, cardiovascular disease, and all-cause mortality) in the general population (17, 22, 23); and second, exploring its correlation with chronic complications (such as carotid atherosclerosis and osteoporosis) in individuals with established DM (24, 25). However, research specifically targeting the key population of individuals with IFG is still relatively scarce. More specifically, no study to date has specifically investigated how the baseline ZJU index influences the bidirectional glycemic outcome trajectories of the IFG population. Existing research has predominantly focused on the unidirectional “disease progression” (i.e., from normoglycemia to DM), often overlooking the clinically more pertinent “healthy reversion” (i.e., from IFG reversion to normoglycemia). Given the significant individual heterogeneity in the degree of IR and compensatory capacity within the IFG population, elucidating whether the ZJU index—a biomarker integrating both hepatic and systemic metabolic information—can effectively identify “prone-to-reversion” favorable subpopulations or “prone-to-progression” high-risk subpopulations remains a critical knowledge gap in the current field of metabolic disease prevention and management.

As a simple computational tool integrating multi-dimensional clinical information, the ZJU index can more accurately assess the risk of metabolic diseases. However, its association with glycemic outcomes—including reversion to normoglycemia and progression to DM—within the specific high-risk population with baseline IFG remains unclear, and there are currently no relevant international reports on this topic. This study aims to, based on a large-scale, multi-center Chinese retrospective cohort, systematically investigate the association of the baseline ZJU index for glycemic status transitions in individuals with IFG and to further explore its potential non-linear relationships and threshold effects. The findings are expected to provide novel perspectives and tools for the early risk stratification and individualized management of patients with IFG.

## Method

### Data source

The raw data utilized in this study are stored in Dryad, an internationally renowned open-access data repository. This dataset originates from a study publicly released by Chen et al. in 2018 (26), and its complete data files are accessible via the unique identifier (https://doi.org/10.5061/dryad.ft8750v). These data were initially collected from the electronic health record system established by the Rich Healthcare Group in China. This system consecutively captured longitudinal clinical monitoring information from individuals undergoing health screenings between 2010 and 2016, encompassing data from all examinees across 32 sites in 11 cities in China. The original study protocol received formal approval from the Ethics Review Committee of the Rich Healthcare Group in China prior to its implementation. In accordance with the data management policy of the Dryad platform, the original researchers have waived all copyright and related ownership of the data. This allows subsequent researchers to reuse and conduct in-depth analysis of the data for non-commercial academic purposes, provided that the terms of its license are adhered to.

### Study population

**Figure 1:**
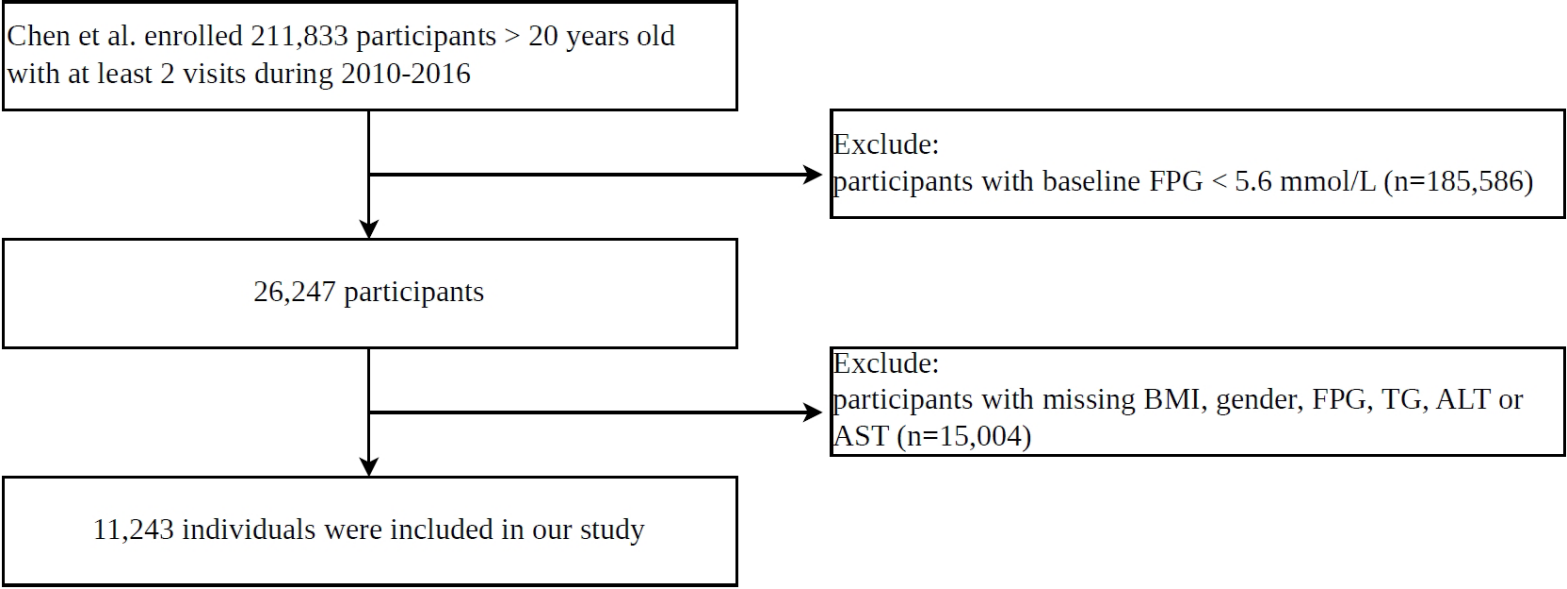
The detailed participant selection flowchart of this study.

This study is a secondary analysis conducted based on the original research by Chen et al. The original study enrolled a total of 211,833 participants who underwent at least two health screenings between 2010 and 2016. The present analysis restricted the study population to individuals with IFG at baseline, with a focus on examining the association of the baseline ZJU index on glycemic status transitions. Based on predefined exclusion criteria, we excluded individuals with a baseline FPG lower than 5.6 mmol/L, as well as those with missing data for BMI, gender, AST, ALT, FPG, or TG. Ultimately, a total of 11,243 participants were included in this analysis. The detailed participant selection flowchart is presented in **Figure 1**.

### Definitions of the exposure variable

The primary exposure variable in this study was the ZJU index, which was treated as a continuous variable. It was estimated using the formula proposed in the literature (18).The ZJU index was calculated as BMl (kg/m^2^) + FPG (mmol/L) + TG (mmol/L) + 3 * ALT/AST ratio, with an additional 2 for females.

### Definitions of the outcome variable

The primary endpoint of this study was defined as the transition of glycemic status, specifically encompassing the following two scenarios: (1) reversion to normoglycemia and (2) progression to incident DM. The adjudication of the aforementioned glycemic statuses was based on the diagnostic criteria recommended by the American Diabetes Association (ADA). Specifically, an individual was classified as having normal fasting glucose if their FPG concentration at the last health examination was below 5.6 mmol/L and​ they self-reported no history of DM. A DM event was defined as having an FPG level of ≥ 7.0 mmol/L at the final examination or a self-reported physician diagnosis of DM. Furthermore, the study follow-up duration was defined as the entire observation period spanning from the date of the first health screening to the date of the last health examination.

### Covariates

The variables encompassed in this study were primarily categorized into five groups: (1) demographic variables, including gender and age; (2) anthropometric indicators, involving height, body weight, and blood pressure (BP); (3) biochemical parameters, encompassing FPG, total cholesterol (TC), TG, low-density lipoprotein cholesterol (LDL-C), high-density lipoprotein cholesterol (HDL-C), ALT, AST, blood urea nitrogen (BUN), and serum creatinine (Scr); (4) lifestyle information, comprising smoking status and drinking status; and (5) family history of DM.

Regarding data acquisition, demographic data, lifestyle information, and family medical history were collected using a uniformly designed structured questionnaire. Height, body weight, and blood pressure data were measured by systematically trained and certified staff following standardized operating procedures. BMI was calculated as body weight in kilograms divided by the square of height in meters. Smoking and drinking status were categorized into three groups—current, former, and never—according to the definitions used in the original database. Venous blood samples were collected by professional personnel from all study participants after fasting for at least 10 hours. The aforementioned biochemical parameters were quantitatively measured using a Beckman Coulter AU5800 automated biochemical analyzer.

### Missing data processing

In handling missing values within the dataset, this study strictly adhered to the standard analytical procedures for observational research. Among the included variables, a total of 8 contained missing data, comprising 6 continuous variables and 2 categorical variables. The specific proportions of missing data are detailed in **Supplementary Table 1**. To minimize potential selection bias or estimation bias resulting from missing data, we performed imputation for the following biochemical parameters with missing values: BUN, Scr, systolic blood pressure (SBP), diastolic blood pressure (DBP), HDL-C, and LDL-C. Specifically, the multiple imputation by chained equations (MICE) method was employed using the “mice” package in R software, performing five iterations of imputation (27) to generate five complete datasets. This method is a common and robust strategy for handling data with arbitrary missing patterns. For missing records in the two categorical variables—smoking and drinking status—we uniformly coded them into a “Not recorded” group. To evaluate the reliability of the multiple imputation method and test the robustness of the analytical results, the primary regression analysis results reported in the main text are based on the pooled dataset after imputation. To further comprehensively verify the stability of the conclusions, this study provides, in the **Supplementary Materials**, the analysis results obtained using only the original complete observation data (pre-imputation) and the data after deleting participants with incomplete covariate information. The direction and strength of the association between the ZJU index and glycemic status transition were largely consistent across the three analytical approaches (post-imputation, pre-imputation, and after deleting cases with missing covariates), indicating that the main findings of this study are not sensitive to the method of handling missing data and that the results possess good stability.

### Statistical method

**Figure 2:**
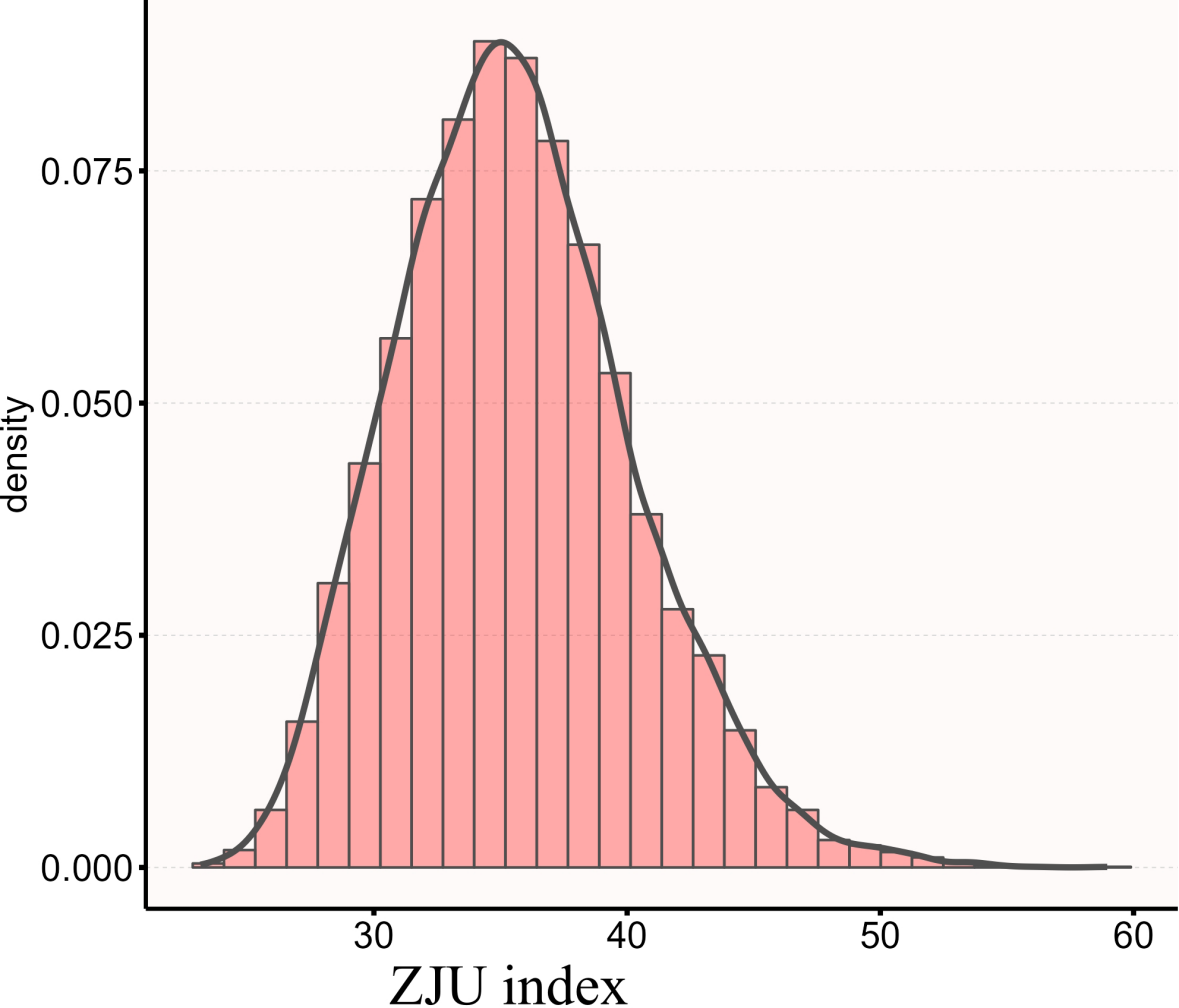
The distribution of ZJU index.

The ZJU index calculated in this study followed a normal distribution, with the distribution across all study participants illustrated in **Figure 2**. Based on quartiles of the ZJU index, all participants were categorized into four groups: Q1 ( ≤ 32.296), Q2 (≤ 35.296), Q3 ( ≤ 38.48), and Q4 ( ≤ 58.959). The normality of continuous variables was assessed using the Kolmogorov-Smirnov test. Variables conforming to a normal distribution are presented as mean ± standard deviation, while those not normally distributed are described as median (interquartile range) [M (IQR)]. Categorical variables are expressed as frequency (percentage) [n (%)]. For between-group comparisons, one-way analysis of variance (ANOVA) was used for normally distributed continuous variables with homogeneity of variance; otherwise, the Kruskal-Wallis H test was employed. Categorical variables were compared using the chi-square test.

Kaplan-Meier curves were used to visualize the association between the ZJU index and glycemic status transition. Differences in transition across different ZJU index quartile groups were statistically analyzed using the log-rank test. This study employed multivariable Cox proportional hazards regression models to assess the association between the calculated ZJU index and glycemic status transition. While controlling for potential confounders, hazard ratios (HRs) and 95% confidence intervals (95% CIs) were estimated. Specifically, two progressively adjusted models were constructed: Model I adjusted for age, gender, SBP, DBP, and family history of DM at baseline; Model II adjusted for age, gender, SBP, DBP, LDL-C, HDL-C, TC, BUN, Scr, smoking status, drinking status, and family history of DM at baseline. To verify the robustness of the relationship between the ZJU index and glycemic status transition from multiple perspectives, this study also performed analyses using multivariable logistic regression models to estimate odds ratios (ORs) and 95% CIs. The sets of adjustment variables included in these models were identical to those in the aforementioned Cox models, allowing for a comprehensive assessment of the consistency of the associations. Consistent statistical conclusions from both regression models would significantly enhance the credibility of the findings. To further explore potential non-linear associations between the ZJU index and glycemic status transition, this study employed restricted cubic splines (RCS) to fit smoothed function curves. When a non-linear trend was suggested, piecewise regression models were utilized to identify potential threshold effects or inflection points. Furthermore, to evaluate the stability of the relationship between the ZJU index and glycemic status transition across different population characteristics, stratified Cox regression analyses were performed, with stratification by variables including gender, age, BMI, SBP, DBP, smoking status, and drinking status. To control for residual confounding, analyses within each subgroup were uniformly adjusted for a set of covariates including age, gender, SBP, DBP, LDL-C, HDL-C, TC, BUN, Scr, smoking status, drinking status, and family history of DM at baseline (except for the stratification variable itself).

### Statistical software

All results are reported in accordance with the STROBE statement. Statistical analyses were performed in the R environment (version 4.2.2; https://www.R-project.org/) and the Free Statistics Analysis Platform (version 2.1.1; https://www.clinicalscientists.cn/freestatistics). A two-tailed *P*-value of less than 0.05 was considered statistically significant. As the present study constitutes a secondary analysis of pre-existing data, no formal *a priori* sample size calculation was performed.

## Result

### Baseline characteristics of the study population

This study included a total of 11,243 individuals with IFG at baseline. **Table 1** presents the demographic and clinical characteristics of the study population stratified by baseline ZJU index quartiles. For the overall population, the mean ZJU index was 35.59 ± 4.64, the mean age was 50.06 ± 14.02 years, males accounted for 7,518 (66.87%) participants, 4,680 (41.63%) reverted to normoglycemia, 5,252 (46.71%) remained in the IFG state, and 1,131 (11.66%) progressed to DM, with a median follow-up of 2.89 years.

**Table 1:** Baseline demographic and clinical characteristics of the study cohort, stratified by ZJU index quartiles.

Variables	Total (n = 11,243)	ZJU index				
		Q1 (n = 2,811)	Q2 (n = 2,810)	Q3 (n = 2,811)	Q4 (n = 2,811)	*P*-Value
ZJU index, Mean ± SD	35.59 ± 4.64	30.00 ± 1.72	33.87 ± 0.86	36.79 ± 0.90	41.72 ± 2.85	< 0.001
Age(years), Mean ± SD	50.06 ± 14.02	47.65 ± 14.99	51.84 ± 14.07	51.67 ± 13.52	49.08 ± 13.00	< 0.001
Gender, n (%)						< 0.001
Male	7518 (66.87)	1395 (49.63)	1776 (63.2)	2058 (73.21)	2289 (81.43)	
Female	3725 (33.13)	1416 (50.37)	1034 (36.8)	753 (26.79)	522 (18.57)	
BMI(kg/m^2^), Mean ± SD	24.84 ± 3.34	20.98 ± 1.58	23.86 ± 1.22	25.78 ± 1.44	28.72 ± 2.59	< 0.001
Height(cm), Mean ± SD	166.66 ± 8.42	164.83 ± 8.31	165.94 ± 8.41	167.14 ± 8.37	168.71 ± 8.09	< 0.001
Body weight(kg), Mean ± SD	69.25 ± 12.12	57.16 ± 7.11	65.82 ± 7.03	72.13 ± 7.54	81.87 ± 10.26	< 0.001
SBP(mmHg), Mean ± SD	127.68 ± 17.81	122.23 ± 17.07	127.26 ± 18.01	129.01 ± 17.36	132.23 ± 17.31	< 0.001
DBP(mmHg), Mean ± SD	78.48 ± 11.22	74.51 ± 10.52	77.60 ± 10.94	79.58 ± 10.99	82.21 ± 10.99	< 0.001
Baseline FPG(mmol/L), Mean ± SD	5.96 ± 0.33	5.87 ± 0.27	5.93 ± 0.31	5.98 ± 0.34	6.06 ± 0.36	< 0.001
TC(mmol/L), Mean ± SD	4.99 ± 0.95	4.73 ± 0.89	4.97 ± 0.93	5.03 ± 0.93	5.23 ± 0.99	< 0.001
TG(mmol/L), M(IQR)	1.40 (0.95, 2.12)	0.90 (0.67, 1.22)	1.28 (0.93, 1.71)	1.60 (1.15, 2.26)	2.29 (1.59, 3.44)	< 0.001
HDL-C(mmol/L), Mean ± SD	1.50 ± 0.37	1.60 ± 0.33	1.51 ± 0.38	1.46 ± 0.36	1.44 ± 0.38	< 0.001
LDL-C(mmol/L), Mean ± SD	3.65 ± 1.43	3.50 ± 1.38	3.64 ± 1.40	3.67 ± 1.42	3.78 ± 1.49	< 0.001
ALT(U/L), M(IQR)	21.80 (15.10, 32.50)	14.60 (11.50, 19.00)	19.30 (15.00, 25.10)	25.00 (18.80, 34.00)	35.80 (25.30, 53.30)	< 0.001
AST(U/L), M(IQR)	24.00 (20.00, 29.40)	21.80 (18.00, 25.65)	23.00 (19.60, 27.20)	24.40 (21.00, 29.90)	28.00 (23.00, 35.95)	< 0.001
BUN(mmol/L), Mean ± SD	5.91 ± 2.97	5.79 ± 3.01	5.95 ± 2.99	5.97 ± 2.92	5.92 ± 2.95	0.108
Scr(μmol/L), Mean ± SD	74.19 ± 16.41	69.84 ± 15.65	73.97 ± 16.85	75.65 ± 15.82	77.31 ± 16.38	< 0.001
Smoking status, n (%)						< 0.001
Current smoker	776 (6.90)	130 (4.62)	172 (6.12)	193 (6.87)	281 (10)	
Ever smoker	164 (1.46)	33 (1.17)	49 (1.74)	40 (1.42)	42 (1.49)	
Never smoker	2493 (22.17)	713 (25.36)	629 (22.38)	594 (21.13)	557 (19.82)	
Not recorded	7810 (69.47)	1935 (68.84)	1960 (69.75)	1984 (70.58)	1931 (68.69)	
Drinking status, n (%)						< 0.001
Current drinker	165 (1.47)	28 (1)	38 (1.35)	41 (1.46)	58 (2.06)	
Ever drinker	659 (5.86)	123 (4.38)	161 (5.73)	166 (5.91)	209 (7.44)	
Never drinker	2609 (23.21)	725 (25.79)	651 (23.17)	620 (22.06)	613 (21.81)	
Not recorded	7810 (69.47)	1935 (68.84)	1960 (69.75)	1984 (70.58)	1931 (68.69)	
Family history of DM, n (%)						< 0.001
No	10977 (97.63)	2757 (98.08)	2726 (97.01)	2768 (98.47)	2726 (96.98)	
Yes	266 (2.37)	54 (1.92)	84 (2.99)	43 (1.53)	85 (3.02)	
Outcomes, n (%)						< 0.001
Normoglycemia	4680 (41.63)	1561 (55.53)	1216 (43.27)	1031 (36.68)	872 (31.02)	
IFG	5252 (46.71)	1138 (40.48)	1354 (48.19)	1391 (49.48)	1369 (48.7)	
DM	1311 (11.66)	112 (3.98)	240 (8.54)	389 (13.84)	570 (20.28)	

As the ZJU index quartile increased (from Q1 to Q4), significant increasing trends were observed in height, body weight, BMI, SBP, DBP, baseline FPG, TC, TG, LDL-C, ALT, AST, and Scr levels (all *P* < 0.001). Age differed significantly across quartiles (*P* < 0.001), but did not show a monotonic increase. Conversely, HDL-C levels showed a significant decreasing trend (*P* < 0.001). Furthermore, the proportions of males, current smokers, and current drinkers also increased with higher ZJU index quartiles (all *P* < 0.001). Regarding glycemic outcomes, a higher ZJU index was associated with a lower proportion of reversion to normoglycemia and a higher proportion of progression to DM, with statistically significant differences across groups (*P* < 0.001).

**Table 2** presents the baseline characteristics of the population stratified by glycemic status at the follow-up endpoint.​ Compared to individuals who remained in the IFG state and those who reverted to normoglycemia, those who ultimately progressed to DM had significantly higher baseline ZJU index, age, proportion of males, BMI, body weight, SBP, DBP, FPG, TC, TG, ALT, and AST levels (all *P* < 0.001), as well as significantly lower HDL-C levels (*P* < 0.001). Significant differences were also observed among the three groups in LDL-C, height, Scr, smoking status, drinking status, and family history of DM (*P* < 0.05).

**Table 2:** Baseline demographic and clinical characteristics of the study cohort, stratified by glycemic status.

Variables	Reversion to normoglycemia(n = 4680)	Persistence of IFG(n = 5252)	Progression to DM(n = 1311)	*P*-value
ZJU index, Mean ± SD	34.60 ± 4.62	35.88 ± 4.43	37.99 ± 4.44	< 0.001
Age(years), Mean ± SD	46.28 ± 13.98	52.03 ± 13.59	55.66 ± 12.29	< 0.001
Gender, n (%)				< 0.001
Male	3027 (64.68)	3558 (67.75)	933 (71.17)	
Female	1653 (35.32)	1694 (32.25)	378 (28.83)	
BMI(kg/m^2^), Mean ± SD	24.22 ± 3.32	25.05 ± 3.24	26.16 ± 3.32	< 0.001
Height(cm), Mean ± SD	166.95 ± 8.47	166.44 ± 8.33	166.46 ± 8.55	0.008
Body weight(kg), Mean ± SD	67.79 ± 12.16	69.65 ± 11.75	72.81 ± 12.61	< 0.001
SBP(mmHg), Mean ± SD	124.30 ± 16.79	129.41 ± 17.88	132.83 ± 18.83	< 0.001
DBP(mmHg), Mean ± SD	76.68 ± 10.71	79.44 ± 11.26	81.05 ± 11.84	< 0.001
Baseline FPG(mmol/L), Mean ± SD	5.83 ± 0.24	5.99 ± 0.32	6.29 ± 0.38	< 0.001
TC(mmol/L), Mean ± SD	4.92 ± 0.94	5.03 ± 0.95	5.07 ± 0.97	< 0.001
TG(mmol/L), M(IQR)	1.27 (0.87, 1.90)	1.45 (1.00, 2.20)	1.73 (1.20, 2.60)	< 0.001
HDL-C(mmol/L), Mean ± SD	1.53 ± 0.36	1.49 ± 0.36	1.45 ± 0.43	< 0.001
LDL-C(mmol/L), Mean ± SD	3.72 ± 1.45	3.60 ± 1.38	3.63 ± 1.51	< 0.001
ALT(U/L), M(IQR)	20.15 (14.10, 31.00)	22.00 (16.00, 32.00)	26.00 (18.00, 40.00)	< 0.001
AST(U/L), M(IQR)	23.60 (19.60, 29.00)	24.00 (20.00, 29.00)	26.00 (21.00, 32.55)	< 0.001
BUN(mmol/L), Mean ± SD	5.92 ± 3.10	5.93 ± 2.92	5.76 ± 2.68	0.153
Scr(μmol/L), Mean ± SD	73.59 ± 16.48	74.71 ± 16.13	74.28 ± 17.25	0.003
Smoking status, n (%)				< 0.001
Current smoker	345 (7.37)	342 (6.51)	89 (6.79)	
Ever smoker	81 (1.73)	63 (1.2)	20 (1.53)	
Never smoker	1205 (25.75)	1094 (20.83)	194 (14.8)	
Not recorded	3049 (65.15)	3753 (71.46)	1008 (76.89)	
Drinking status, n (%)				< 0.001
Current drinker	69 (1.47)	74 (1.41)	22 (1.68)	
Ever drinker	328 (7.01)	267 (5.08)	64 (4.88)	
Never drinker	1234 (26.37)	1158 (22.05)	217 (16.55)	
Not recorded	3049 (65.15)	3753 (71.46)	1008 (76.89)	
Family history of DM, n (%)				0.006
No	4566 (97.56)	5146 (97.98)	1265 (96.49)	
Yes	114 (2.44)	106 (2.02)	46 (3.51)	

**Figure 3:**
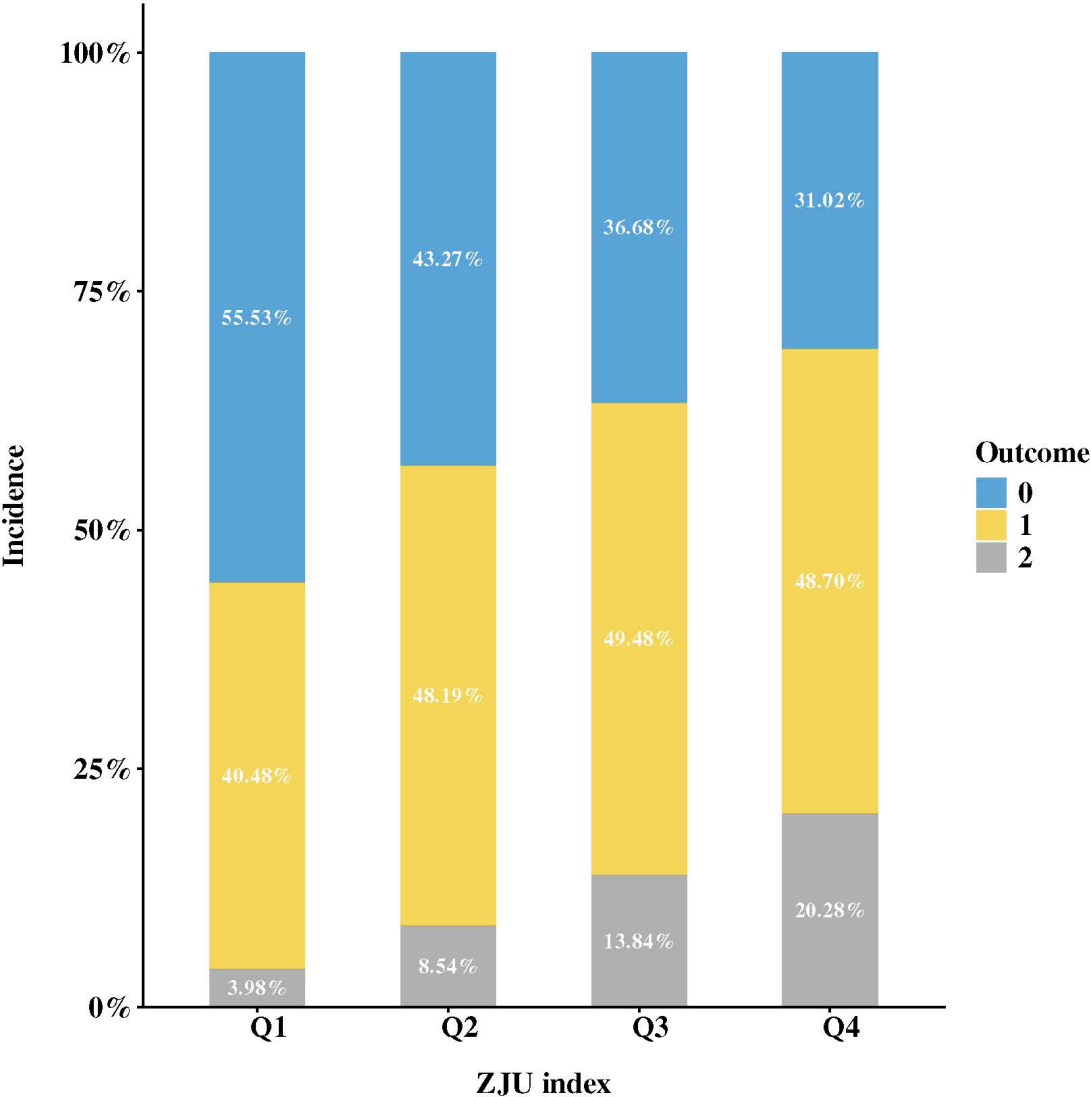
The incidence rate for reversion to normoglycemia/progression to DM stratified by ZJU index quartiles.

As shown in **Figure 3**,​ a stacked bar chart visually displays the distribution proportions of different glycemic transition outcomes in the study population grouped by baseline ZJU index quartiles. As the ZJU index quartile increased, the proportion of the blue section (labeled 0) representing reversion to normoglycemia gradually decreased, while the proportion of the gray section (labeled 2) representing progression to DM gradually increased. This visual result is consistent with the data distribution in the “Outcomes” row of **Table 1**.

### Association Between ZJU index and glycemic status transition

As shown in **Table 3**, in the Cox regression analysis evaluating the association between the ZJU index and the risk of glycemic status transition, the ZJU index was negatively associated with the risk of reversion to normoglycemia and positively associated with the risk of progression to DM.

**Table 3:** ​Relationship of ZJU index with glucose status transition in an IFG population assessed by Cox proportional-hazards regression in multiple models.

Variables	Crude model		Model I		Model II	
	HR (95%CI)	*P*-value	HR (95%CI)	*P*-value	HR (95%CI)	*P*-value
IFG to normoglycemia						
ZJU index	0.95 (0.94, 0.95)	< 0.001	0.96 (0.95, 0.97)	< 0.001	0.97 (0.96, 0.97)	< 0.001
(ZJU index quartiles)						
Q1	1.00 (Reference)		1.00 (Reference)		1.00 (Reference)	
Q2	0.74 (0.68, 0.79)	< 0.001	0.84 (0.78, 0.91)	< 0.001	0.92 (0.85, 0.99)	0.031
Q3	0.63 (0.58, 0.68)	< 0.001	0.73 (0.67, 0.79)	< 0.001	0.81 (0.75, 0.88)	< 0.001
Q4	0.52 (0.47, 0.56)	< 0.001	0.59 (0.54, 0.64)	< 0.001	0.65 (0.6, 0.72)	< 0.001
*P* for trend		< 0.001		< 0.001		< 0.001
IFG to DM						
ZJU index	1.1 (1.09, 1.11)	< 0.001	1.11 (1.09, 1.12)	< 0.001	1.12 (1.1, 1.13)	< 0.001
(ZJU index quartiles)						
Q1	1.00 (Reference)		1.00 (Reference)		1.00 (Reference)	
Q2	1.98 (1.58, 2.48)	< 0.001	1.76 (1.41, 2.21)	< 0.001	1.96 (1.56, 2.45)	< 0.001
Q3	3.27 (2.65, 4.03)	< 0.001	2.98 (2.4, 3.68)	< 0.001	3.37 (2.72, 4.18)	< 0.001
Q4	4.62 (3.77, 5.65)	< 0.001	4.37 (3.55, 5.38)	< 0.001	5.14 (4.16, 6.35)	< 0.001
*P* for trend		< 0.001		< 0.001		< 0.001

Crude model: we did not adjust for other covariates. Model I: adjusted for age, gender, SBP, DBP, and family history of DM at baseline. Model II: further adjusted for LDL-C, HDL-C, TC, BUN, Scr, smoking status, and drinking status at baseline.

In the Cox regression analysis evaluating the association between the ZJU index and glycemic status transition, a one-unit increase in the ZJU index was associated with a significantly lower risk of reversion to normoglycemia (crude model HR = 0.95, 95% CI: 0.94–0.95). This negative association remained significant in fully adjusted model (Model II HR = 0.97, 95% CI: 0.96–0.97). Compared to the lowest quartile (Q1), participants in higher ZJU index quartiles showed progressively lower reversal risks in the fully adjusted model (Q2 HR = 0.92; Q3 HR = 0.81; Q4 HR = 0.65; all *P* < 0.05), with a significant dose-response trend (*P* for trend < 0.001). Conversely, each one-unit increase in the ZJU index was associated with a higher risk of progression to DM (crude model HR = 1.1, 95% CI: 1.09–1.11). This positive association was strengthened in adjusted models (Model II HR = 1.12, 95% CI: 1.10–1.13). Using Q1 as reference, the risk of progression increased substantially across quartiles in Model II (Q2 HR = 1.96; Q3 HR = 3.37; Q4 HR = 5.14; all *P* < 0.001), with a significant dose-response trend (*P* for trend < 0.001).

As shown in **Table 4**, the results of the logistic regression analysis were consistent with those from the Cox regression, further confirming the significant association between the ZJU index and both states of glycemic transition. The ZJU index was negatively associated with the probability of reversion to normoglycemia (Model II OR = 0.93, 95% CI: 0.92–0.94, *P* < 0.001). Compared with the Q1 group, the ORs for reversion to normoglycemia in the Q2, Q3, and Q4 groups in the fully adjusted model were 0.73, 0.57, and 0.41, respectively (all *P* < 0.001). For progression to DM, the ZJU index showed a strong positive association (Model II OR = 1.14, 95% CI: 1.13–1.16, *P* < 0.001). Compared with the Q1 group, the ORs for progression to DM in the Q2, Q3, and Q4 groups in the fully adjusted model were 1.92, 3.39, and 5.94, respectively (all *P* < 0.001).

**Table 4:** ​Relationship of ZJU index with glycemic status transition in an IFG population assessed by logistic regression in multiple models.

Variables	Crude model		Model I		Model II	
	OR (95%CI)	*P*-value	OR (95%CI)	*P*-value	OR (95%CI)	*P*-value
IFG to normoglycemia						
ZJU index	0.92 (0.91, 0.93)	< 0.001	0.93 (0.92, 0.94)	< 0.001	0.93 (0.92, 0.94)	< 0.001
(ZJU index quartiles)						
Q1	1.00(Reference)		1.00 (Reference)		1.00 (Reference)	
Q2	0.61 (0.55, 0.68)	< 0.001	0.72 (0.64, 0.8)	< 0.001	0.73 (0.65, 0.82)	< 0.001
Q3	0.46 (0.42, 0.52)	< 0.001	0.55 (0.49, 0.62)	< 0.001	0.57 (0.5, 0.63)	< 0.001
Q4	0.36 (0.32, 0.4)	< 0.001	0.41 (0.36, 0.46)	< 0.001	0.41 (0.37, 0.47)	< 0.001
*P* for trend		< 0.001		< 0.001		< 0.001
IFG to DM						
ZJU index	1.13 (1.11, 1.14)	< 0.001	1.14 (1.12, 1.15)	< 0.001	1.14 (1.13, 1.16)	< 0.001
(ZJU index quartiles)						
Q1	1.00(Reference)		1.00 (Reference)		1.00 (Reference)	
Q2	2.25 (1.79, 2.83)	< 0.001	1.94 (1.53, 2.45)	< 0.001	1.92 (1.52, 2.43)	< 0.001
Q3	3.87 (3.11, 4.81)	< 0.001	3.4 (2.73, 4.25)	< 0.001	3.39 (2.71, 4.25)	< 0.001
Q4	6.13 (4.97, 7.56)	< 0.001	5.8 (4.66, 7.22)	< 0.001	5.94 (4.75, 7.43)	< 0.001
*P* for trend		< 0.001		< 0.001		< 0.001

Crude model: we did not adjust for other covariates. Model I: adjusted for age, gender, SBP, DBP, and family history of DM at baseline.Model II: further adjusted for LDL-C, HDL-C, TC, BUN, Scr, smoking status, and drinking status at baseline.

### Kaplan-Meier curves for glycemic status transition stratified by the ZJU index

**Figure 4:**
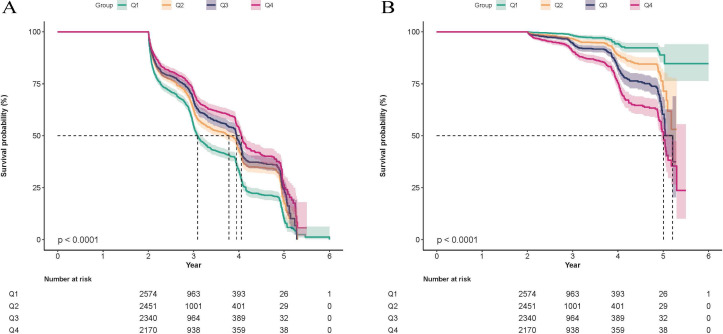
Kaplan-Meier curves for glycemic status transition stratified by the ZJU index quartiles. **(A)**​ Reversion to normoglycemia. **(B)**​ Progression to DM.

**Figure 4** presents the Kaplan-Meier curves stratified by ZJU index quartiles. **Figure 4A** depicts the survival curve for reversion to normoglycemia, and **Figure 4B** depicts the curve for progression to DM. The results show that the Q1 group had the highest probability of reversion to normoglycemia and the lowest probability of progression to DM, whereas the Q4 group had the lowest probability of reversion and the highest probability of progression. Log-rank tests indicated that the differences in the probabilities of reversion to normoglycemia (**Figure 4A**) and progression to DM (**Figure 4B**) among the different ZJU index quartile groups were statistically significant (*P* < 0.001).

### Dose-response relationship between the ZJU index and risks of glycemic status transition

**Figure 5:**
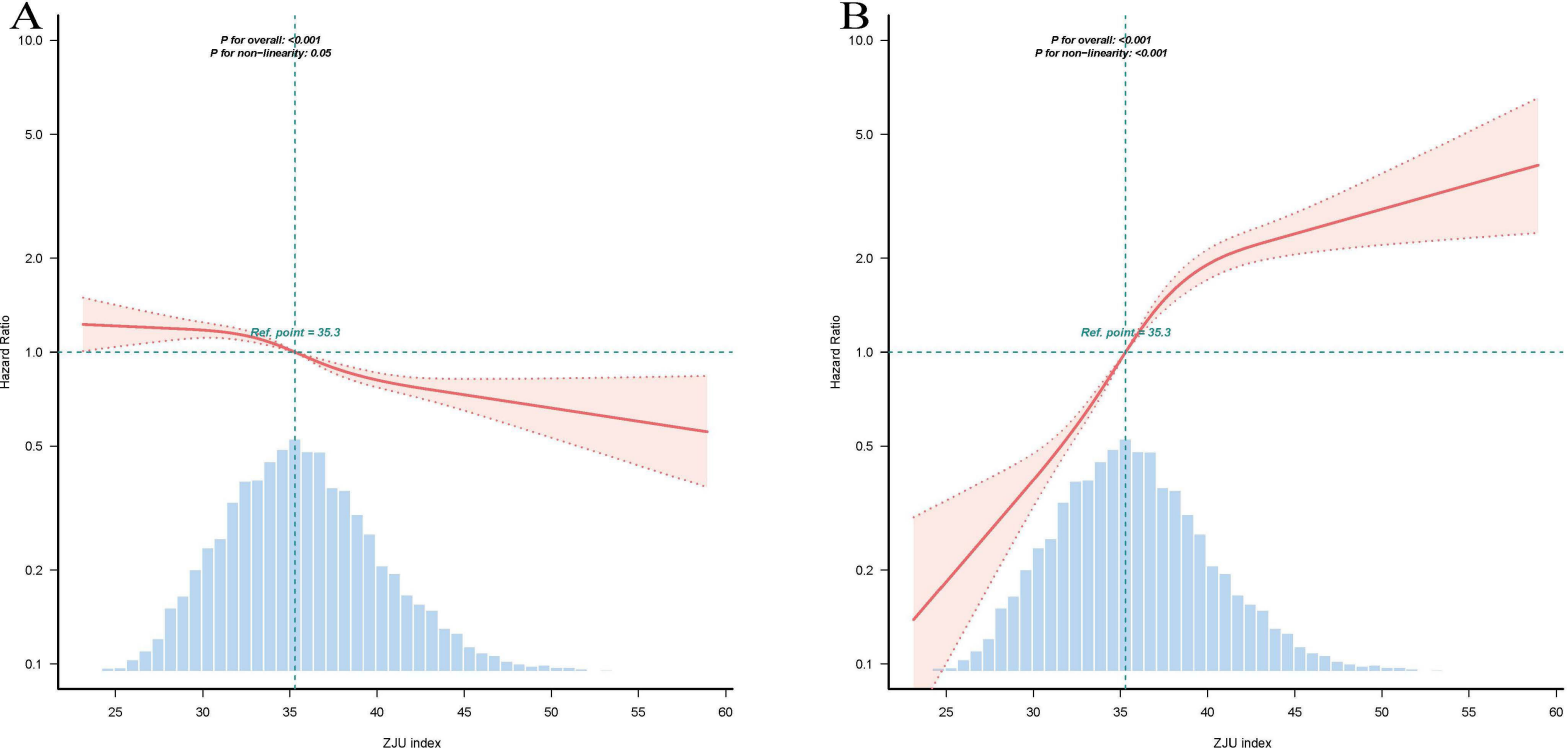
The relationship between ZJU index and reversion to normoglycemia/progression to DM in an IFG population. Further adjusted for for age, gender, SBP, DBP, LDL-C, HDL-C, TC, BUN, Scr, smoking status and family history of DM at baseline. **(A)** Association between the ZJU index and reversion to normoglycemia. **(B)** Association between the ZJU index and progression to DM.

The dose-response relationship between the ZJU index and the risk of glycemic outcomes was further analyzed using RCS models (**Figure 5**). **Figure 5A** shows the association between the ZJU index and the risk of reversion to normoglycemia in the fully adjusted model. The curve presents as a gently declining straight line (*P* for non-linearity = 0.05), steadily decreasing with increasing ZJU index, supporting a linear inverse association between the ZJU index and the risk of reversion to normoglycemia. **Figure 5B** shows the association between the ZJU index and the risk of progression to DM in the fully adjusted model. The curve displays a distinct non-linear characteristic (*P* for non-linearity < 0.001). At lower ZJU index levels, the risk of progression to DM increased rapidly with the index; after the ZJU index exceeded a certain threshold, the increasing trend in the risk of progression to DM with a rising index plateaued. This supports a non-linear dose-response relationship between the ZJU index and the risk of progression to DM.

### Threshold effect of the ZJU index on the risk of progression to DM

To further evaluate the non-linear relationship between the ZJU index and the risk of DM progression, a threshold effect analysis was conducted (**Table 5**). The results indicated a non-linear association between the ZJU index and the risk of DM progression. Using 38.499 as the inflection point, piecewise linear regression revealed that when the ZJU index was < 38.499, each one-unit increase was associated with a 20.3% higher progression risk (HR = 1.203, 95% CI: 1.168–1.239, *P* < 0.001). When the ZJU index was ≥ 38.499, the increasing trend in risk attenuated, with each one-unit increase associated with a 5.0% higher risk (HR = 1.050, 95% CI: 1.016–1.086, *P* = 0.004).

**Table 5:** Threshold effect analysis of ZJU index on progression to DM in an IFG population.

Variables	HR(95%CI)	*P*-Value
IFG to DM		
< 38.499	1.203 (1.168, 1.239)	< 0.001
≥ 38.499	1.05 (1.016, 1.086)	0.004
*P* for log-likelihood ratio test		<0.001

Further adjusted for age, gender, SBP, DBP, LDL-C, HDL-C, TC, BUN, Scr, smoking status and family history of DM at baseline.

### Subgroup analyses of the association between the ZJU index and glycemic status transition

**Figure 6:**
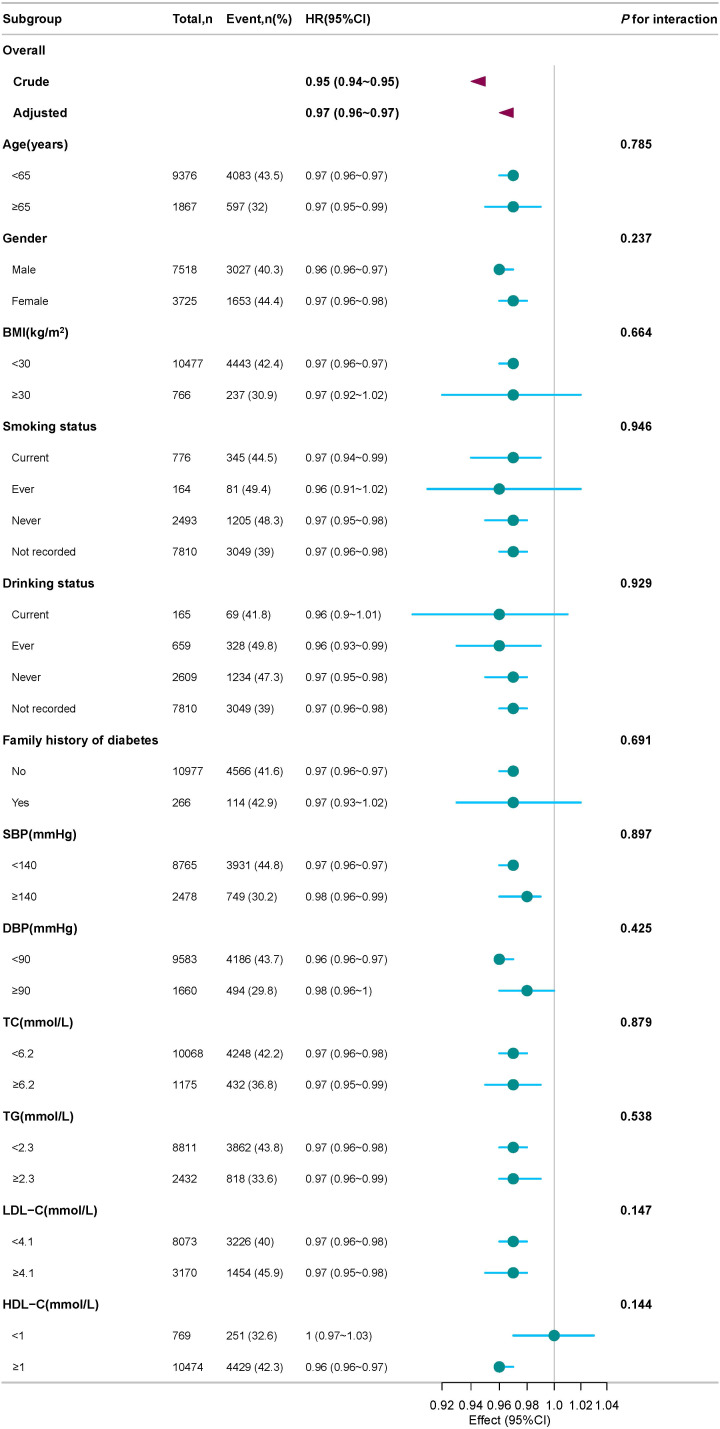
Forest plot of subgroup analysis for the association of the ZJU index with reversion to normoglycemia in an IFG population.

**Figure 6** presents the forest plot of subgroup analyses for the association between the ZJU index and the risk of reversion to normoglycemia.​ Across all predefined subgroups—including those stratified by age, gender, BMI, SBP, DBP, TG, TC, LDL-C, HDL-C, smoking status, drinking status, and the presence or absence of a family history of DM—the ZJU index consistently showed an inverse association with the risk of reversion. The HRs for all subgroup analyses were less than 1, which is entirely consistent in direction with the negative association between the ZJU index and the risk of reversion to normoglycemia observed in the primary analysis (**Table 3**, Model II).

**Figure 7:**
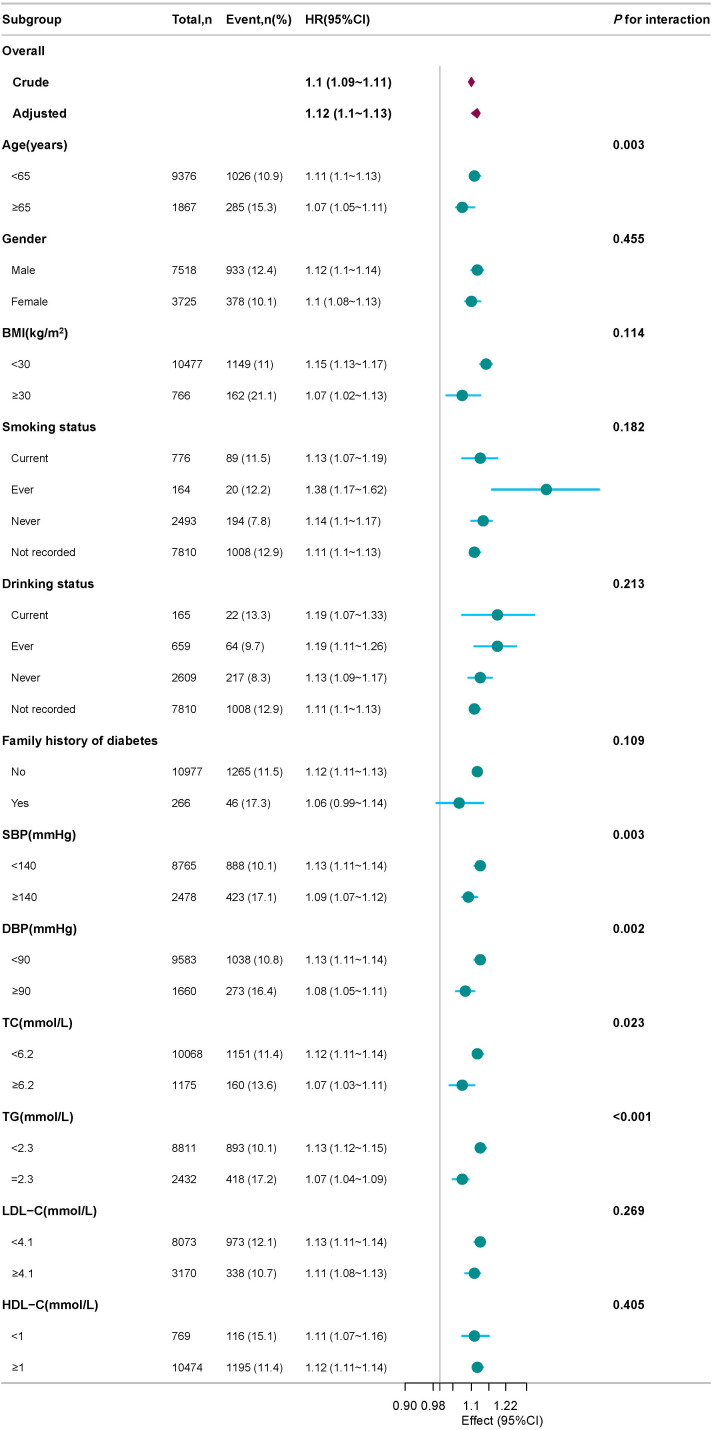
Forest plot of subgroup analysis for the association of the ZJU index with progression to DM in an IFG population.

**Figure 7** presents the forest plot of subgroup analyses for the association between the ZJU index and the risk of progression to DM.​ In all predefined subgroups, the ZJU index consistently showed a positive association with the risk of DM progression. The HRs for all subgroup analyses were greater than 1, which is entirely consistent in direction with the observation from the primary analysis (**Table 3**, Model II) that the ZJU index is a risk factor for DM progression.

### Sensitivity analyses

To assess the robustness of the main findings, we conducted two sensitivity analyses. First, Cox proportional hazards regression and logistic regression analyses were repeated on the original dataset containing all baseline covariates, including those with missing values (**Supplementary Tables 2**, **3**). The results showed that in the multivariable-adjusted model (Model II), the direction and significance of the associations between the ZJU index and glycemic outcomes were consistent with the primary analyses (**Tables 3**, **4**). Specifically, the ZJU index was significantly and inversely associated with the risk of reversion to normoglycemia (Cox regression: HR = 0.97, 95% CI: 0.95–0.98, *P* < 0.001; Logistic regression: OR = 0.92, 95% CI: 0.90–0.94, *P* < 0.001) and significantly and positively associated with the risk of progression to DM (Cox regression: HR = 1.14, 95% CI: 1.10–1.19, *P* < 0.001; Logistic regression: OR = 1.15, 95% CI: 1.10–1.19, *P* < 0.001). Second, to exclude potential bias from missing data, we re-performed the analyses after removing individuals with any missing baseline covariates (**Supplementary Tables 4**, **5**). In this complete-case dataset, the direction of association, statistical significance, and the dose-response trends based on quartiles (all *P* for trend < 0.001) between the ZJU index and both glycemic outcomes remained unchanged. Compared to the primary analysis, the magnitude of the effect estimates (HRs/ORs) showed stronger associations. For instance, in the Cox Model II adjusted for the most covariates, the HR for progression to DM in the Q4 group versus the Q1 group increased to 6.39 (95% CI: 3.23–12.62); in the corresponding Logistic Model II, the OR for the Q4 group increased to 8.25 (95% CI: 4.02–16.94).

## Discussion

This study evaluated the association between the ZJU index and the direction of glycemic transition in a large-scale, multi-center Chinese population with IFG using retrospective cohort data. Through a comprehensive analysis of the obtained data, we derived clear and robust conclusions. The main findings encompass the following four aspects. First, the baseline ZJU index is strongly and independently associated with glycemic outcomes in individuals with IFG. Specifically, a higher ZJU index was significantly associated with a reduced probability of reversion to normoglycemia and an increased risk of progression to incident DM. These associations persisted after full adjustment for a range of traditional confounding factors. Second, we observed significant dose-response relationships, whereby each unit increase in the ZJU index corresponded to a diminished protective effect for reversion and an augmented risk for DM progression, reinforcing its reliability as a continuous risk marker. Third, notably, a distinct non-linear association was identified between the ZJU index and the risk of DM progression, with a key threshold pinpointed (inflection point at 38.499). Below this threshold, the DM risk escalated sharply with increasing ZJU index; above it, the risk entered a relatively high plateau phase. This finding suggests a potential critical value for the ZJU index that may serve as a reference for clinical decision-making. Fourth, extensive subgroup analyses demonstrated that the aforementioned associations were consistently present and directionally uniform across subgroups stratified by age, gender, BMI, SBP, DBP, TG, TC, LDL-C, HDL-C, smoking status, drinking status, and the presence or absence of a family history of DM, confirming the robustness and generalizability of the results. Furthermore, to assess the robustness of our conclusions, we conducted in-depth sensitivity analyses. By employing multiple imputation to handle missing data and comparing results from three analytical approaches—using the original complete data (pre-imputation), the imputed data, and the data after deleting cases with missing covariates—the direction and magnitude of the association between the ZJU index and glycemic status transition remained largely consistent. This series of analyses confirmed that the main findings of this study are not sensitive to the method of handling missing data, demonstrating good stability. This study confirms that the ZJU index, a composite measure easily obtainable in clinical practice, can effectively stratify risk within the vast high-risk IFG population and simultaneously evaluate their likelihood of transitioning toward two opposing outcomes: “reversion” or “progression.” To our knowledge, this is the first cohort study conducted in a large-scale Chinese IFG population that comprehensively evaluates the long-term risk of baseline ZJU index for bidirectional glycemic transition—namely, reversion to normoglycemia and progression to DM. Our findings provide a novel and concise quantitative tool for precise risk stratification and early intervention in the prediabetes stage.

This study confirms that the ZJU index is a powerful clinical indicator associated with glycemic outcomes in individuals with IFG, a finding highly consistent with the pathophysiological mechanism of the ZJU index as a composite marker of metabolic dysregulation. Since its introduction, the ZJU index has been used to identify IR and MASLD. Its components—BMI, FPG, TG, and the ALT/AST ratio—quantify obesity, hyperglycemia, dyslipidemia, and hepatic steatosis/inflammation, respectively, all pointing to IR as the core pathophysiological driver of prediabetes progression (22, 24). Consequently, a higher baseline ZJU index signifies a more severe state of systemic IR and metabolic dysregulation. On this pathological basis, individuals with IFG find it more difficult to revert to normoglycemia and are more prone to progress to DM, which aligns entirely with the natural history of metabolic disease progression. This is also biologically consistent with the index’s established role in predicting incident DM risk in the general population (22, 23). The unique value of this study lies in extending the application scenario of the ZJU index from “disease risk prediction” to “clinical outcome risk stratification.” Previous research has primarily focused on its use in the general population or individuals with established DM to predict disease onset (e.g., DM, cardiovascular disease) or assess comorbidity risk (e.g., MASLD) (22, 23, 28, 29). In contrast, this study is the first to precisely target the critical intervention window of “prediabetes”—the IFG population. Risk stratification within this specific group holds more urgent clinical significance: assessing whether an individual is more likely to “revert” or “progress” provides clinicians with more direct and personalized decision-making guidance than predicting long-term onset in the general population. This marks an important advancement in the application of the ZJU index from an “epidemiological risk identifier” to a “bedside prognostic tool.” Furthermore, this study innovatively constructs a “bidirectional analytical framework” model, simultaneously assessing the probabilities of progression to DM or reversion to normoglycemia in individuals with IFG. This approach overcomes the limitation of existing prognostic studies focusing solely on unidirectional outcomes, providing a more comprehensive risk assessment tool for clinical decision-making. While most studies focus only on a single negative outcome (e.g., progression to DM), we uniquely evaluated the ZJU index’s association with both “reversion” (a positive outcome) and “progression” (a negative outcome), thereby offering a more complete risk panorama. This enables the ZJU index not only to identify “high-risk” individuals requiring intensive intervention but also to pinpoint those with “high reversal potential,” contributing valuable information for refined, individualized management strategies for prediabetes. Notably, we identified a non-linear association and a clear threshold effect between the ZJU index and the risk of DM progression. This finding provides a potential quantitative cutoff for clinical practice. Similar to thresholds reported for the ZJU index in other health outcomes, such as 40.6 for predicting gallstone risk (19) or 38.87 for MASLD risk in individuals with DM (28), the threshold identified here may signify a critical state of metabolic dysregulation. Below this threshold, DM risk increases sharply with a rising index, suggesting this phase might be the most cost-effective “golden window” for intervention. Exceeding this threshold indicates that an individual has entered a high-risk plateau, likely signifying severely compromised metabolic homeostasis, necessitating the initiation of more aggressive clinical management. This offers preliminary grounds for future exploration into transforming the ZJU index from a continuous variable into a categorical one with clear clinical action points. In summary, this study not only validates the bidirectional risk stratification utility of the ZJU index in an IFG population but also, by focusing on this key population, constructing a bidirectional model, and identifying a risk threshold, deepens its clinical applicability. It provides new evidence for precise risk stratification and management in the prediabetes stage.

The significant association between the ZJU index and glycemic outcomes observed in this study can be reasonably explained by the pathophysiological mechanisms corresponding to its individual components. The ZJU index integrates metrics for obesity, FPG, blood lipids, and hepatic enzymes, which collectively represent a core pathogenic triad—inflammation, IR, and metabolic dysregulation. The pathophysiological basis of this strong association is primarily manifested in the following three intertwined dimensions. First, IR is the central mechanism throughout. Obesity, FPG, hypertriglyceridemia, and abnormal liver enzymes are all key manifestations of IR across different organs and metabolic levels (20). A high BMI, particularly visceral fat accumulation, leads to the release of excessive free fatty acids and pro-inflammatory factors from adipose tissue, interfering with insulin signaling in skeletal muscle and the liver. Elevated FPG is a direct consequence of insufficient insulin action, while high TG is a typical feature of dyslipidemia in the context of IR. An increased ALT/AST ratio often indicates hepatic steatosis, which is itself closely associated with hepatic IR (30). Therefore, a higher ZJU index signifies a more severe state of systemic IR. In this state, pancreatic β-cells are forced to compensate by secreting more insulin to maintain glucose homeostasis, leading to an increased long-term burden and gradual functional decline. This makes it more difficult for individuals with IFG to naturally revert to normoglycemia and significantly increases their risk of progressing to DM. Second, hepatic steatosis plays a pivotal role in the deterioration of glucose metabolism. The ALT/AST ratio, as part of the ZJU index, is closely associated with the degree of hepatic fat infiltration and serves as a simple indicator for identifying MASLD (28). As the central organ for glucose and lipid metabolism, a steatotic liver directly induces and exacerbates hepatic IR. This leads to abnormally active hepatic gluconeogenesis and reduced glycogen synthesis, resulting in the continuous output of glucose into the circulation. This process constitutes a direct pathway linking hepatic fat to systemic glucose dysregulation, explaining why the hepatic fat indicator within the ZJU index is closely linked to DM progression. Third, chronic low-grade inflammation forms a vicious cycle that exacerbates metabolic dysregulation. Obesity and hepatic steatosis are not only consequences of metabolic abnormalities but also sources of chronic low-grade inflammation (31). Enlarged adipose tissue, especially visceral fat, and steatotic hepatocytes secrete large amounts of pro-inflammatory cytokines (e.g., tumor necrosis factor-α, interleukin-6) and adipokines. This persistent inflammatory state can further impair tyrosine phosphorylation of insulin receptor substrate (IRS) proteins, blocking the downstream PI3K-Akt signaling pathway, thereby aggravating IR in peripheral tissues and the liver (32, 33). Consequently, a positive feedback loop is formed among inflammation, IR, and metabolic abnormalities (hyperglycemia, dyslipidemia), making the state of metabolic dysregulation represented by a high ZJU index more entrenched and naturally steering glycemic outcomes toward an unfavorable direction. As a composite clinical indicator, the risk stratification efficacy of the ZJU index stems from its simultaneous reflection of abnormal states in adipose tissue, the liver, and systemic glucose/lipid metabolism. These abnormalities mutually amplify each other through the common pathway of IR, collectively disrupting glucose homeostasis. The findings of this study support this view. What the ZJU index quantifies is precisely the overall burden of this multisystem, progressive metabolic dysregulation, and the magnitude of this burden directly determines the direction of the glycemic trajectory in individuals with IFG.

The findings of this study provide readily translatable insights for the clinical management of prediabetes, specifically IFG. First, the ZJU index, as a composite measure easily derived from routine health check-up data, provides an efficient tool for risk stratification within the IFG population. Its ease of use facilitates adoption in primary care settings, helping clinicians identify IFG subgroups with “high reversal potential” (low ZJU index) and those at “high progression risk,” thereby enabling targeted intervention and optimal allocation of healthcare resources. Second, the non-linear threshold identified in this study offers a clear evidence-based basis for intensive intervention strategies. Individuals with a ZJU index above this threshold should be considered at very high risk for DM prevention, warranting not only intensified lifestyle modification but also consideration for early pharmacological intervention (34). For those with a low index, standard lifestyle guidance combined with psychological support may suffice for risk management. Finally, dynamic changes in the ZJU index theoretically hold promise as a comprehensive surrogate endpoint for assessing intervention efficacy in future studies. Given its ability to concurrently reflect improvements across multiple metabolic dimensions, this index could offer a more holistic gauge of metabolic benefit than any single parameter. However, this potential application was not evaluated in the current study, as changes in cardiometabolic parameters were not assessed as outcomes.

This study possesses several significant methodological strengths. First, leveraging a large-scale, multi-center health screening cohort of Chinese adults, we enrolled a substantial number of individuals with IFG. The sample has a broad geographic distribution, enhancing the representativeness of our findings for the Chinese population. Second, the study is a retrospective cohort study utilizing pre-existing health screening data. All baseline data were derived from standardized health examinations, and the follow-up duration was sufficiently long to reliably capture long-term glycemic status transition events among individuals with IFG. Regarding statistical methodology, we conducted comprehensive and rigorous analyses. We not only applied Cox proportional hazards regression models but also supplemented the assessment of glycemic status transition with multivariable logistic regression models. This approach jointly verified the robustness of the association between the ZJU index and the outcomes from different statistical perspectives. To further ensure the reliability of the conclusions, we utilized RCS to explore and visualize potential non-linear relationships between the exposure and outcomes. We handled missing data in covariates using multiple imputation, thereby reducing potential bias arising from data absence. Furthermore, extensive subgroup analyses and a series of sensitivity analyses were performed. The results consistently showed that the primary associations remained stable across different subgroups and data handling methods, strengthening the generalizability and robustness of our findings.

However, several limitations of this study should be considered when interpreting the results. The primary limitation lies in its observational design. Although we controlled for known confounders as much as possible through multivariable adjustment and advanced statistical methods, the influence of residual or unmeasured confounding (e.g., detailed dietary patterns, intensity of physical activity, use of specific medications) on the observed associations cannot be completely ruled out. Therefore, a causal relationship between the ZJU index and glycemic outcomes cannot be established. Secondly, the study data primarily relied on routine health examinations. While this ensured the easy accessibility of the metrics, it may have introduced some degree of measurement error. More importantly, the determination of glycemic status transition (reversion, persistence, progression) in this study was based solely on FPG and self-reported DM diagnosis, without using the oral glucose tolerance test (OGTT) or HbA1c as supplementary diagnostic criteria. Using FPG alone for DM diagnosis has lower sensitivity than OGTT and may fail to identify some individuals with DM or prediabetes who present only with elevated postprandial glucose or elevated HbA1c. This could lead to an underestimation of the disease progression rate and may have some impact on the accuracy of the risk assessment. Furthermore, an inherent mathematical coupling exists between the ZJU index and our defined outcomes. Because FPG is a core variable in the calculation of the ZJU index, and progression to DM is diagnosed via an FPG threshold, baseline FPG is undeniably a strong, direct driver of the observed outcomes. We acknowledge that the ZJU index should not be viewed merely as a tool intended to mathematically outperform FPG in predicting incident hyperglycemia. Instead, its true clinical utility lies in metabolic subphenotyping. While FPG alone reflects an instantaneous state of glucose dysregulation, it cannot differentiate the underlying pathophysiological mechanisms. By incorporating BMI, TG, and the ALT/AST ratio, the ZJU index captures the concurrent burden of systemic adiposity, lipotoxicity, and hepatic insulin resistance. Therefore, the ZJU index serves as a complementary stratification tool, identifying a specific IFG subphenotype characterized by a greater burden of obesity and hepatic insulin resistance, which may guide more comprehensive, mechanism-targeted interventions beyond simple glycemic control. At the same time, the baseline data for this cohort were collected between 2010 and 2016. While the fundamental pathophysiological mechanisms linking obesity, hepatic insulin resistance, and glycemic transitions remain biologically constant, population demographics and lifestyle factors have evolved over the past decade. Although the escalating modern prevalence of metabolic syndrome arguably makes the ZJU index even more clinically relevant today, the exact quantitative risk thresholds derived from this historical cohort should be interpreted with caution when applied to contemporary populations. Future prospective studies utilizing recent cohorts are necessary to validate these findings for present-day clinical practice. Finally, the study population was predominantly of Han ethnicity. Given the differences in genetic background, lifestyle, and disease risk among different races/ethnicities, caution is warranted when extrapolating our findings to other ethnic groups. Future validation in multi-ethnic cohorts is needed.

Based on the findings and limitations of this study, future research could consider the following three directions. First, prospective interventional studies, such as randomized controlled trials (RCTs), should be conducted to verify whether a risk-stratified management strategy based on the ZJU index can effectively improve glycemic outcomes in individuals with IFG. Concurrently, methods such as Mendelian randomization could be employed to further investigate the potential causal relationship. Second, the risk stratification utility of this index needs to be validated under more rigorous diagnostic conditions that incorporate the OGTT and HbA1c. Furthermore, exploring the use of dynamically changing ZJU index values or combining it with emerging biomarkers (e.g., adiponectin) may lead to the development of models with superior clinical stratification capability. Finally, external validation in cohorts of different races, ethnicities, and healthcare settings is crucial for establishing its generalizability and clinical applicability.

## Conclusion

In the population with IFG, the baseline ZJU index serves as an independent and effective composite indicator strongly associated with the future direction of glycemic transition. Specifically, a higher ZJU index exhibits a linear negative association with reversion to normoglycemia and a non-linear positive association with progression to incident DM. The ZJU index may serve as a practical tool for risk stratification and precision management in the IFG population, thereby guiding preventive strategies for individuals at high risk of developing DM.

## Data Availability

Publicly available datasets were analyzed in this study. This data can be found here: The dataset presented in this study is available in the online repository (https://doi.org/10.5061/dryad.ft8750v).

## Glossary

DM: Diabetes mellitus
IDF: International Diabetes Federation
IFG: Impaired fasting glucose
HbA1c: Glycated hemoglobin A1c
IR: Insulin resistance
FPG: Fasting plasma glucose
BMI: Body mass index
TG: Triglycerides
ALT: Alanine aminotransferase
AST: Aspartate aminotransferase
ZJU index: Zhejiang University Index
FLI: Fatty liver index
HIS: Hepatic steatosis index
MASLD: Metabolic dysfunction-associated steatotic liver disease
VAI: Visceral adiposity index
ADA: American Diabetes Association
TC: Total cholesterol
LDL-C: Low-density lipoprotein cholesterol
HDL-C: High-density lipoprotein cholesterol
BUN: Blood urea nitrogen
Scr: Serum creatinine
MICE: Multiple imputation by chained equations
IQR: Interquartile range
ANOVA: Analysis of variance
HR: Hazard ratio
CI: Confidence interval
OR: Odds ratio
RCS: Restricted cubic splines
SD: Standard deviation
SBP: Systolic blood pressure
DBP: Diastolic blood pressure
OGTT: Oral glucose tolerance test
RCTs: Randomized controlled trials
IRS: Insulin receptor substrate
PI3K-Akt: Phosphoinositide 3-kinase - Protein kinase B
STROBE: Strengthening the Reporting of Observational Studies in Epidemiology
