# Supplementary material for: Association between ZJU index and glycemic outcomes in individuals with impaired fasting glucose: a retrospective multicenter Chinese cohort study
Source: Front Endocrinol (Lausanne). 2026 Apr 23;17:1811341. doi: 10.3389/fendo.2026.1811341 (PMC13149108; doi:10.3389/fendo.2026.1811341)
Supplement: Supplementary file 1 [file SupplementaryFile1.docx]

**Supplementary Table 1 Description of missing data.**

| **Variables** | **Non-missing** | **Missing** | **Miss percentage(%)** |
| --- | --- | --- | --- |
| Age | 11243 | 0 | 0 |
| Gender | 11243 | 0 | 0 |
| Height | 11243 | 0 | 0 |
| Body weight | 11243 | 0 | 0 |
| BMI | 11243 | 0 | 0 |
| BaseFPG | 11243 | 0 | 0 |
| ALT | 11243 | 0 | 0 |
| AST | 11243 | 0 | 0 |
| BUN | 10001 | 1242 | 11.0469 |
| Scr | 10745 | 498 | 4.4294 |
| SBP | 11238 | 5 | 0.0445 |
| DBP | 11238 | 5 | 0.0445 |
| HDL-C | 7166 | 4077 | 36.2626 |
| LDL-C | 7726 | 3517 | 31.2817 |
| TC | 11243 | 0 | 0 |
| TG | 11243 | 0 | 0 |
| Family histroy of Diabetes | 11243 | 0 | 0 |
| Drinking | 3433 | 7810 | 69.4654 |
| Smoking | 3433 | 7810 | 69.4654 |

**Supplementary Table 2 Relationship of ZJU index with** **glycemic status conversion in an IFG population assessed by Cox proportional-hazards regression in multiple models (the original dataset).**

| Variables | Crude model | | Model Ⅰ | | Model Ⅱ | |
| --- | --- | --- | --- | --- | --- | --- |
|  | HR (95%CI) | *P*-Value | HR (95%CI) | *P*-Value | HR (95%CI) | *P*-Value |
| IFG to normoglycemia | | |  |  |  |  |
| ZJU index | 0.95 (0.94, 0.95) | < 0.001 | 0.96 (0.95, 0.97) | < 0.001 | 0.97 (0.95, 0.98) | < 0.001 |
| (ZJU index quartiles) |  |  |  |  |  |  |
| Q1 | 1.00 (Reference) |  | 1.00 (Reference) |  | 1.00 (Reference) |  |
| Q2 | 0.74 (0.68, 0.79) | < 0.001 | 0.84 (0.78, 0.91) | < 0.001 | 0.82 (0.69, 0.98) | 0.028 |
| Q3 | 0.63 (0.58, 0.68) | < 0.001 | 0.73 (0.67, 0.79) | < 0.001 | 0.7 (0.58, 0.85) | < 0.001 |
| Q4 | 0.52 (0.47, 0.56) | < 0.001 | 0.59 (0.54, 0.64) | < 0.001 | 0.63 (0.51, 0.78) | < 0.001 |
| *P* for trend |  | < 0.001 |  | < 0.001 |  | < 0.001 |
| IFG to DM | | |  |  |  |  |
| ZJU index | 1.1 (1.09, 1.11) | < 0.001 | 1.11 (1.09, 1.12) | < 0.001 | 1.14 (1.1, 1.19) | < 0.001 |
| (ZJU index quartiles) |  |  |  |  |  |  |
| Q1 | 1.00 (Reference) |  | 1.00 (Reference) |  | 1.00 (Reference) |  |
| Q2 | 1.98 (1.58, 2.48) | < 0.001 | 1.76 (1.41, 2.21) | < 0.001 | 2.39 (1.17, 4.9) | 0.017 |
| Q3 | 3.27 (2.65, 4.03) | < 0.001 | 2.98 (2.4, 3.68) | < 0.001 | 4.17 (2.09, 8.33) | < 0.001 |
| Q4 | 4.62 (3.77, 5.65) | < 0.001 | 4.36 (3.54, 5.38) | < 0.001 | 6.38 (3.23, 12.58) | < 0.001 |
| *P* for trend |  | < 0.001 |  | < 0.001 |  | < 0.001 |

Crude model: we did not adjust other covariates.
Model I: adjusted for age, gender, SBP, DBP and family history of diabetes at baseline.

Model II: further adjusted for LDL-C, HDL-C, TC, BUN, Scr, smoking status, and drinking status at baseline.

**Supplementary Table 3 ​Relationship of ZJU index with glycemic status conversion in an IFG population assessed by Logistic regression in multiple models** **(the original dataset).**

| Variables | Crude model | | Model Ⅰ | | Model Ⅱ | |
| --- | --- | --- | --- | --- | --- | --- |
|  | OR (95%CI) | *P*-Value | OR (95%CI) | *P*-Value | OR (95%CI) | *P*-Value |
| IFG to normoglycemia | | |  |  |  |  |
| ZJU index | 0.92 (0.91, 0.93) | < 0.001 | 0.93 (0.92, 0.94) | < 0.001 | 0.92 (0.9, 0.94) | < 0.001 |
| (ZJU index quartiles) |  |  |  |  |  |  |
| Q1 | 1.00 (Reference) |  | 1.00 (Reference) |  | 1.00 (Reference) |  |
| Q2 | 0.61 (0.55, 0.68) | < 0.001 | 0.72 (0.64, 0.8) | < 0.001 | 0.54 (0.41, 0.72) | < 0.001 |
| Q3 | 0.46 (0.42, 0.52) | < 0.001 | 0.55 (0.49, 0.62) | < 0.001 | 0.44 (0.32, 0.59) | < 0.001 |
| Q4 | 0.36 (0.32, 0.4) | < 0.001 | 0.41 (0.36, 0.46) | < 0.001 | 0.33 (0.24, 0.45) | < 0.001 |
| *P* for trend |  | < 0.001 |  | < 0.001 |  | < 0.001 |
| IFG to DM | | |  |  |  |  |
| ZJU index | 1.13 (1.11, 1.14) | < 0.001 | 1.14 (1.12, 1.15) | < 0.001 | 1.15 (1.1, 1.19) | < 0.001 |
| (ZJU index quartiles) |  |  |  |  |  |  |
| Q1 | 1.00 (Reference) |  | 1.00 (Reference) |  | 1.00 (Reference) |  |
| Q2 | 2.25 (1.79, 2.83) | < 0.001 | 1.93 (1.53, 2.44) | < 0.001 | 2.88 (1.38, 6.02) | 0.005 |
| Q3 | 3.87 (3.11, 4.81) | < 0.001 | 3.41 (2.73, 4.25) | < 0.001 | 5.05 (2.45, 10.38) | < 0.001 |
| Q4 | 6.13 (4.97, 7.56) | < 0.001 | 5.78 (4.65, 7.2) | < 0.001 | 8.1 (3.96, 16.58) | < 0.001 |
| *P* for trend |  | < 0.001 |  | < 0.001 |  | < 0.001 |

Crude model: we did not adjust other covariates.
Model I: adjusted for age, gender, SBP, DBP and family history of diabetes at baseline.

Model II: further adjusted for LDL-C, HDL-C, TC, BUN, Scr, smoking status, and drinking status at baseline.

**Supplementary Table 4 Relationship of ZJU index with glycemic status conversion in an IFG population assessed by Cox proportional-hazards regression in multiple models (excluding participants with incomplete covariate dataset).**

| Variables | Crude model | | Model Ⅰ | | Model Ⅱ | |
| --- | --- | --- | --- | --- | --- | --- |
|  | HR (95%CI) | *P*-Value | HR (95%CI) | *P*-Value | HR (95%CI) | *P*-Value |
| IFG to normoglycemia | | |  |  |  |  |
| ZJU index | 0.93 (0.91, 0.94) | < 0.001 | 0.95 (0.94, 0.97) | < 0.001 | 0.97 (0.95, 0.98) | < 0.001 |
| (ZJU index quartiles) |  |  |  |  |  |  |
| Q1 | 1.00 (Reference) |  | 1.00 (Reference) |  | 1.00 (Reference) |  |
| Q2 | 0.58 (0.49, 0.69) | < 0.001 | 0.71 (0.6, 0.85) | < 0.001 | 0.81 (0.68, 0.97) | 0.02 |
| Q3 | 0.48 (0.4, 0.57) | < 0.001 | 0.58 (0.48, 0.7) | < 0.001 | 0.68 (0.56, 0.82) | < 0.001 |
| Q4 | 0.42 (0.35, 0.5) | < 0.001 | 0.53 (0.43, 0.65) | < 0.001 | 0.66 (0.54, 0.82) | < 0.001 |
| *P* for trend |  | < 0.001 |  | < 0.001 |  | < 0.001 |
| IFG to DM | | |  |  |  |  |
| ZJU index | 1.11 (1.07, 1.14) | < 0.001 | 1.12 (1.08, 1.16) | < 0.001 | 1.14 (1.1, 1.19) | < 0.001 |
| (ZJU index quartiles) |  |  |  |  |  |  |
| Q1 | 1.00 (Reference) |  | 1.00 (Reference) |  | 1.00 (Reference) |  |
| Q2 | 2.99 (1.49, 6.01) | 0.002 | 2.39 (1.18, 4.82) | 0.015 | 2.73 (1.35, 5.53) | 0.005 |
| Q3 | 3.6 (1.82, 7.1) | < 0.001 | 3.29 (1.65, 6.57) | 0.001 | 3.97 (1.98, 7.93) | < 0.001 |
| Q4 | 5.58 (2.89, 10.77) | < 0.001 | 5.07 (2.58, 9.96) | < 0.001 | 6.39 (3.23, 12.62) | < 0.001 |
| *P* for trend |  | < 0.001 |  | < 0.001 |  | < 0.001 |

Crude model: we did not adjust other covariates.
Model I: adjusted for age, gender, SBP, DBP and family history of diabetes at baseline.

Model II: further adjusted for LDL-C, HDL-C, TC, BUN, Scr, smoking status, and drinking status at baseline.

**Supplementary Table 5 ​Relationship of ZJU index with glycemic status conversion in an IFG population assessed by Logistic regression in multiple models (excluding participants with incomplete covariate dataset).**

| Variables | Crude model | | Model Ⅰ | | Model Ⅱ | |
| --- | --- | --- | --- | --- | --- | --- |
|  | OR (95%CI) | *P*-Value | OR (95%CI) | *P*-Value | OR (95%CI) | *P*-Value |
| IFG to normoglycemia | | |  |  |  |  |
| ZJU index | 0.91 (0.89, 0.93) | < 0.001 | 0.92 (0.9, 0.94) | < 0.001 | 0.92 (0.9, 0.94) | < 0.001 |
| (ZJU index quartiles) |  |  |  |  |  |  |
| Q1 | 1.00 (Reference) |  | 1.00 (Reference) |  | 1.00 (Reference) |  |
| Q2 | 0.44 (0.34, 0.57) | < 0.001 | 0.53 (0.4, 0.7) | < 0.001 | 0.52 (0.4, 0.69) | < 0.001 |
| Q3 | 0.36 (0.28, 0.46) | < 0.001 | 0.42 (0.32, 0.56) | < 0.001 | 0.42 (0.31, 0.57) | < 0.001 |
| Q4 | 0.3 (0.23, 0.39) | < 0.001 | 0.36 (0.27, 0.49) | < 0.001 | 0.35 (0.26, 0.49) | < 0.001 |
| *P* for trend |  | < 0.001 |  | < 0.001 |  | < 0.001 |
| IFG to DM | | |  |  |  |  |
| ZJU index | 1.14 (1.11, 1.18) | <0.001 | 1.15 (1.11, 1.2) | <0.001 | 1.15 (1.1, 1.19) | <0.001 |
| (ZJU index quartiles) |  |  |  |  |  |  |
| Q1 | 1.00 (Reference) |  | 1.00 (Reference) |  | 1.00 (Reference) |  |
| Q2 | 4.02 (1.98, 8.16) | < 0.001 | 3.2 (1.56, 6.59) | 0.002 | 3.27 (1.58, 6.76) | 0.001 |
| Q3 | 5.56 (2.79, 11.09) | < 0.001 | 4.74 (2.33, 9.62) | < 0.001 | 4.84 (2.35, 9.94) | < 0.001 |
| Q4 | 9.91 (5.08, 19.34) | < 0.001 | 8.47 (4.23, 16.98) | < 0.001 | 8.25 (4.02, 16.94) | < 0.001 |
| *P* for trend |  | < 0.001 |  | 0.01 |  | < 0.001 |

Crude model: we did not adjust other covariates.
Model I: adjusted for age, gender, SBP, DBP and family history of diabetes at baseline.

Model II: further adjusted for LDL-C, HDL-C, TC, BUN, Scr, smoking status, and drinking status at baseline.
